# Perfluoropolymer/Molecular Sieve Mixed-Matrix Membranes

**DOI:** 10.3390/membranes9020019

**Published:** 2019-01-23

**Authors:** Gianni Golemme, Anna Santaniello

**Affiliations:** 1 Department of Environmental and Chemical Engineering, University of Calabria, Via P. Bucci 45 A, 87036 Rende, Italy; 2 Physics Department, University of Calabria, Via P. Bucci 22 C, 87036 Rende, Italy; anna.santaniello@fis.unical.it

**Keywords:** glassy amorphous perfluoropolymers, mixed matrix membranes, zeolitic molecular sieves, gas separation, interfacial compatibilization, fractional free volume, restricted diffusion, barriers to mass transport, four phases Maxwell model, finite element modelling of transport

## Abstract

Despite the outstanding chemical, thermal and transport properties of amorphous and glassy perfluorinated polymers, only few works exist on the preparation and transport properties of perfluoropolymer/molecular sieves mixed-matrix membranes (MMMs), probably because of their poor compatibility. In this review, the compatibilization of ceramic molecular sieves with perfluorinated matrices is considered first, examining the effect of the surface treatment on the gas transport properties of the filler. Then the preparation of the defect-free hybrid membranes and their gas separation capabilities are described. Finally, recent modelling of the gas transport properties of the perfluoropolymer MMMs is reviewed. The systematic use of molecular sieves of different size and shape, either permeable or impermeable, and the calculation of the bulk transport properties of the molecular sieves—i.e., the unrestricted diffusion and permeability—allow to understand the nature of the physical phenomena at work in the MMMs, that is the larger the perfluoropolymer fractional free volume at the interface, and restricted diffusion at the molecular sieves. This knowledge led to the formulation of a new four-phase approach for the modelling of gas transport. The four-phase approach was implemented in the frame of the Maxwell model and also for the finite element simulation. The four-phase approach is a convenient representation of the transport in MMMs when more than one single interfacial effect is present.

## 1. Introduction

Fluoropolymers display better chemical and solvent resistance with respect to similar hydrocarbon polymers, therefore they are widely used to prepare membranes for battery separators, microfiltration and ultrafiltration, membrane contactors and chemical reactors [1,2,3,4,5]. The best thermal, chemical and solvent resistances are obtained in perfluorinated polymers (PFPs), where the strength of C–C and C–F bonds and the shielding of the carbon skeleton by fluorine atoms prevent chemical attacks [6,7,8].

Perfluoropolyacids (e.g., Nafion^®^) are semi-crystalline and hydrophilic PFPs. They are used as proton exchange membranes in fuel cells and for electrolysis, such as in the chlor-alkali process. Perfluoropolyacid-based mixed-matrix membranes (MMMs), i.e., mixtures of a filler in a polymeric matrix [9], were reviewed elsewhere [7]. In this work, instead, the few pioneering examples of MMMs made of molecular sieves embedded in amorphous and glassy PFP matrices will be considered.

Poly(tetrafluoroethylene) [10] is insoluble because of its semi-crystalline nature, but the presence of bulky or atactic substituents on the carbon chain affords amorphous, glassy and hydrophobic polymers which can be dissolved in fluorinated solvents, lending themselves to the preparation of membranes via solvent evaporation. Hydrophobic PFPs are apolar thanks to the symmetry around the carbon atoms, and the strong attraction of F atoms towards all electrons in the molecule minimizes the interactions with light and the polarizability of the material. As a consequence, hydrophobic and amorphous PFPs display very low refractive indexes [11], the only intermolecular interactions are very weak dispersion forces, and their cohesive energy density and solubility parameters are smaller than those of the corresponding hydrocarbon polymers [12,13]. These features correspond to peculiar macroscopic properties of amorphous and hydrophobic PFPs, i.e., no solubility in common solvents, low specific surface energy, hydrophobic and organophobic behavior, poor adhesion of almost any substance, including glues and adhesives. The main difficulty to overcome to prepare hydrophobic PFP-based MMMs is the homogeneous mixing of finely dispersed nano-fillers in the polymer, and a good adhesion at the interface.

**Figure 1:**
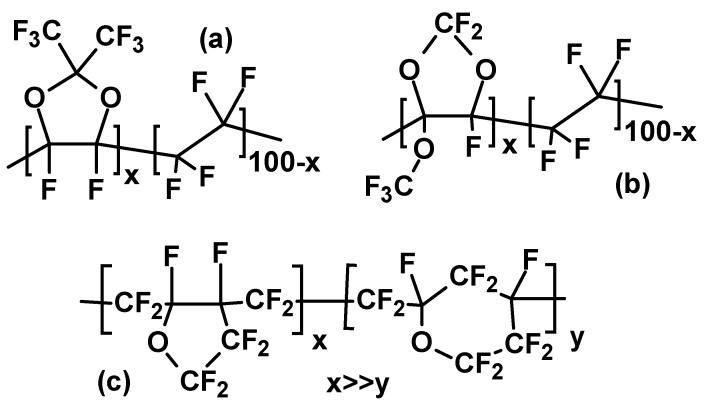
Repeat units of amorphous perfluorinated polymers: (**a**) Teflon^®^ AF, grade 2400: x = 87, grade 1600: x = 65; (**b**) Hyflon^®^ AD, grade 80X: x = 85, grade 60X: x = 60; (**c**) Cytop^®^. *x* and *y* represent the molar composition and do not imply a sequence length.

The first commercial soluble and glassy PFPs appeared about 30 years ago (Figure 1): co-polymers of perfluoro-1,3-dioxoles (Teflon^®^ AF, grades 1600 and 2400, from DuPont; Hyflon^®^ AD, grades 40, 60 and 80, from Ausimont—now Solvay Advanced Polymers) [14], and poly(perfluoro-4-vinyloxy-1-butene), also known with the commercial name Cytop^®^ (Asahi Glass) [11].

Theoretical studies indicate very high potential energy barriers for the rotation around the bonds linking two dioxolane rings in Teflon AF (Figure 1a) and Hyflon AD (Figure 1b). The polymer chains have limited or no rotational freedom, and the stiffness of the backbone increases with the bulkiness (Teflon AF > Hyflon AD, see Figure 1) and the percent of dioxole monomer [15]. In turn, stiffness of the backbone and amount of bulky groups correlate with the glass transition temperature (T_g_), the gas permeability, and the fractional free volume (FFV), i.e., the fraction of the volume which is not occupied by the electronic clouds of the polymer: Teflon AF2400 (T_g_ = 240 °C) > Teflon AF1600 (T_g_ = 160 °C) > Hyflon AD 80 X (T_g_ = 135 °C) > Hyflon AD 60X (T_g_ = 110 °C) > Cytop (T_g_ = 108 °C) [6,16,17]. Teflon^®^ AF 2400, the most permeable polymer of this group, has a free volume of more than 30% [6,18,19], and it is one of the most permeable polymers known today [9].

Gas transport through dense membranes is governed by Equation (1) [20,21]:

*J_i_* = *P_i_*(*p_il_* − *p_i0_*)/*l* (1)

where *J_i_* is the volume (molar) flux of component *i* (e.g., in units cm^3^(STP)/(cm^2^·s)), *l* the membrane thickness, *p_i_*_0_ the partial pressure of component *i* on the feed side, *p_il_* the partial pressure of component *i* on the permeate side, and the permeability *P_i_* represents the ability of molecules *i* to permeate through the membrane material.

The mass transport through dense, non-porous membranes obeys a solution–diffusion mechanism [20,21]. In its simplest form, the permeability *P_i_* of the substance *i* is a function of its solubility in the membrane (*S_i_*, a thermodynamic quantity) and of its diffusivity in the membrane (*D_i_,* a kinetic quantity), as defined in Equation (2):

*P_i_* = *S_i_*·*D_i_* (2)

Selectivity measures a membrane’s ability to separate the components of a mixture. Ideal selectivity (*α_ij_*) is the ratio of permeabilities of pure gases *i* and *j*, and is defined by Equation (3):

*α_ij_* = *P_i_/P_j_* (3)

The combination of Equation (2) and Equation (3) yields Equation (4):

*α_ij_* = (*S_i_*/*S_j_*)·(*D_i_*/*D_j_*) = *α_S_*·*α_D_* (4)

which highlights the fact that the overall permeability selectivity *α_ij_* is the product of the solubility selectivity *α_S_* and of the diffusion selectivity *α_D_*.

The size of a certain molecule (Table 1) governs its mobility in a polymer: the larger the size, the smaller *D*. Instead, the higher its condensability (e.g., as measured by boiling point or critical temperature) the larger its solubility in the polymer. In rubbers, the solubility selectivity dominates over diffusion, because the liquid-like motions of the polymer chains determine a high penetrant mobility and have a leveling effect on the different sizes of the penetrants. The frozen macromolecular motions of glassy polymers instead limit diffusion, and the mobility selectivity dominates over solubility. Therefore, glassy polymers are usually size selective and poorly permeable. However, when the glassy polymer backbone is extremely stiff, and an effective polymer packing cannot be reached, very high FFVs and large permeabilities are observed, such as in certain polyacetylenes [22,23], in the polymers of intrinsic micro-porosity (PIMs) [24], and in glassy amorphous PFPs, as described above.

Glassy amorphous and hydrophobic PFP membranes perform best in those gas separations (He/H_2_, H_2_/hydrocarbons, He/N_2_, He/CO_2_, He/CH_4_, N_2_/CH_4_) where the diffusion selectivity in favour of the smaller molecule is offset, in part, by the solubility selectivity in favour of the tardier, more condensable species [9]. In such cases the modest affinity of the penetrants to PFPs further reduces the unfavorable contribution of solubility to the overall selectivity, when compared to hydrocarbon-based polymers [6]. Another interesting province of PFPs is natural gas sweetening, where CO_2_ is at the same time smaller and more soluble than CH_4_. The outstanding resistance to solvents (aliphatic and aromatic hydrocarbons, water, amines) and to plasticization of Hyflon AD60X is used in commercial high flux membranes for the upgrading of natural gas containing large fractions of CO_2_, that would spoil the separation properties of cellulose acetate or polyimide membranes [5].

**Figure 2:**
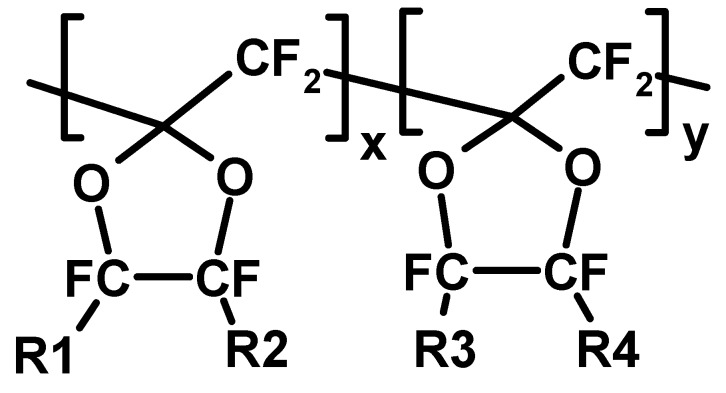
Repeat units of homo- and co-polymers of perfluoro(2-methylene-1,3-dioxolane)s. R1–R4 = F, CF_3_.

In the last few years, other amorphous and glassy PFPs have been synthesized and studied for gas separation: atactic poly(hexafluoropropene) [27], new perfluoro-1,3-dioxoles [28], homo- and co-polymers of perfluoro(2-methylene-1,3-dioxolane)s (Figure 2) [29,30,31]. The latter class of PFPs looks extremely interesting for the preparation of gas separation membranes, with impressive separation factors, beyond the 2008 Robeson upper bound for the H_2_/CH_4_, He/CH_4_, CO_2_/CH_4_, and N_2_/CH_4_ separations [30,31].

In gas separation, the outstanding properties of perfluoropolymers are potentially useful to overcome the limited resistance of hydrocarbon-based polymers, as in the purification of natural gas [32], but unfortunately their intrinsic permselectivity often represents a limit for the potential applications of gas separation membranes [9]. The trade-off between gas permeability and selectivity of polymers can be overcome by using MMMs, i.e., membranes in which small particles of a filler are dispersed in a continuous polymeric phase. Mixed-matrix membranes combine the advantages of both components, since they couple the ease of production and the good mechanical properties of polymeric membranes with the outstanding transport properties of suitable fillers [33].

Fumed silica, titania, and magnesium oxide are among the non-porous fillers used to prepare MMMs. Porous fillers offer an additional degree of freedom for the improvement of the separation capabilities of the polymeric matrices. The porous fillers used for MMMs have been recently reviewed [34]: zeolites, metal-organic frameworks (MOFs), carbon nanotubes, activated carbon, microporous organic polymers, mesoporous oxides, porous bi-dimensional graphene, and graphene oxides, and more. Molecular sieves are crystalline materials with uniform, well-defined micropores (<2 nm), which are able to sort molecules according to their size; they include zeolites, made of SiO^4^ and AlO^4^ tetrahedra, zeotypes containing TO^4^ tetrahedra other than SiO^4^ and AlO^4^ (e.g., PO^4^, GeO^4^, TiO^4^), and analogous non-zeolitic materials containing elements with different coordination number, such as TiO_6_ octahedra in ETS-4 and ETS-10 [34]. In the following, the preparation and the transport properties of MMMs made of molecular sieves dispersed in glassy and hydrophobic PFP is reviewed. First, the strategy for the compatibilization of molecular sieves with the perfluoropolymers is considered. Then the preparation and the separation properties of several MMMs are described. Finally, the macroscopic modelling of the gas transport properties of PFP MMMs is analysed, together with the microscopic features of the membranes. The new four phase approach developed to account for the physical phenomena observed in the PFP MMMs can be used for other MMMs with complex interfacial effects.

## 2. Surface Modification of Molecular Sieves and Surface Permeability

A good adhesion of the glassy PFP to the surface of the molecular sieve is a key factor to avoid the formation of defective gaps at the interface, which would spoil the separation performances of the MMMs, acting as non-selective by-passes for the transport of gases [35]. Unlike rubbers, the achievement of a good adhesion is complicated by the mismatch between the vitrified glassy polymer, which keeps shrinking during the removal of the residual solvent, and the rigid zeolitic molecular sieve. It has to be considered that hydrophilic hydroxyls covering the outer surface of the molecular sieves [36] strongly repel hydrophobic PFPs and perfluorinated solvents.

The several strategies used to disperse molecular sieves in glassy, hydrocarbon-based polymers —polysulfones, polyimides, polybenzimidazoles, etc.—rely on the formation of the strongest possible interactions between the two phases. Covalent bonds form when the dangling 3-aminopropyl groups of zeolites treated with 3-aminopropylalkoxysilanes open and react with the imide rings of polyimides. The covalent bonds strongly facilitate the adhesion of the polymer and the production of defect-free polymer-filler interfaces [37]. In other cases, hydrogen bonds or polar interactions are intense enough to guarantee an intimate contact between the two phases. The strongly polar surface of zeolite 4A is able to adhere to the hydrophobic but highly polarizable poly(1-trimethylsilyl-1-propyne) [38]. Unfortunately, hydrophobic PFPs are chemically inert, apolar, and not polarizable, therefore the strategy was chosen to make the zeolitic molecular sieves fluorophilic, via a post-synthetic grafting of perfluorinated alkyl tails.

The reaction of a moderate excess of 1H,1H,2H,2H-perfluorodecyltrichlorosilane with the external −OH groups of the molecular sieves (e.g., silicalite-1, SAPO-34) made the water contact angle increase from 89° to 150° [39]. The control on the reaction parameters (e.g., dry conditions, low temperature, hydrophobic solvents, short reaction times) produced a continuous mono-layer of vertically aligned perfluorinated moieties tightly packed on the outer surface of the molecular sieves, as indicated by the results of X-ray photoelectron spectroscopy [40]. The modified sieve crystals exposed the terminal −CF_3_ groups of the grafts, and their surface properties became comparable to those of Teflon particles, as indicated by the formation of homogeneous suspensions in perfluorinated solvents. The practically unchanged BET surface area (e.g., 611 vs. 616 m^2^·g^−1^ before the grafting of SAPO-34 [41]) indicated that the external modification did not plug the pores of the particles.

**Figure 3:**
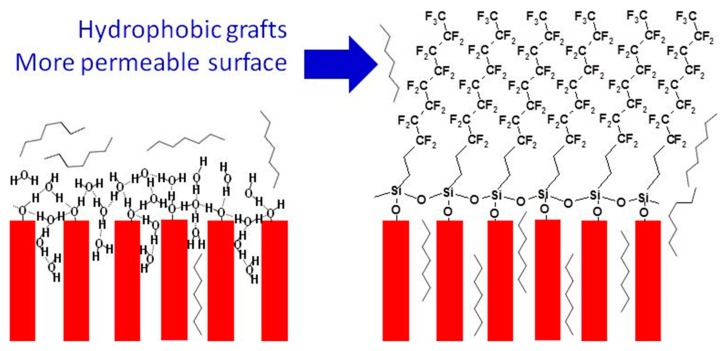
Pictorial representation of the sorption of n-heptane at the surface of silicalite-1 before and after the modification with 1H,1H,2H,2H-perfluorodecyltrichlorosilane. Reproduced from Reference [40] with permission. Copyright of Elsevier B.V.

The effect of the external grafting on the transport of penetrants in and out of silicalite-1 crystals was tested with H_2_ at cryogenic temperatures [36], and with *n*-heptane at room temperature [40]. The first relevant result of the experiments was that the rate of sorption in both cases depended on the modification of the outer surface. This can only happen if the surface permeability is the rate determining step in adsorption, i.e., that barriers for the transport of mass exist on the outer surface of silicalite-1, and that the sorption/desorption rates of species of different size (H_2_, *n*-heptane), at different temperatures, was determined by the presence of these barriers. The second surprising result was that the bulky 1H,1H,2H,2H-perfluorodecyl tails on the surface enhanced the sorption/desorption rates of both H_2_ and *n*-heptane with respect to the as-made molecular sieve. The explanation of this effect was the reduction of the hydrophilic character of the outer surface, where the terminated crystal framework, rich of silanol ≡Si–OH groups, promotes the adsorption of moisture (Figure 3). This hypothesis was supported by a theoretical study predicting, at room temperature, the formation of a water layer on the surface of silicalite-1, extending into the silicalite pores for about 1.0–1.5 nm [41].

## 3. Preparation of Hydrophobic PFP/Molecular Sieve MMMs

**Scheme 1:**
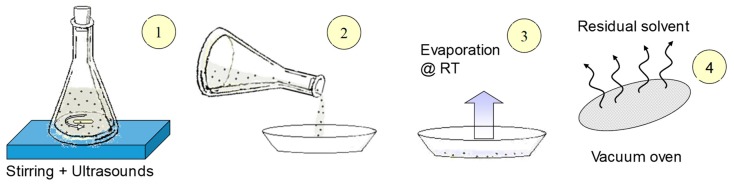
Sequences in the preparation of self-supported perfluorinated polymer (PFP)/molecular sieve MMMs.

As made zeolitic molecular sieves are usually impermeable, because their pores are filled with the templates, the organic substances directing their synthesis. At this stage, their BET area was measured, in order to determine the stoichiometric amount of the modification agent needed [40]. Then the sieves were calcined to free their pores from the organics. If the size of the crystals was small, they were first embedded in a cross-linked network of an organic polymer. The larger average distance among them greatly reduced the sintering into aggregates [42]. Also, as made, impermeable molecular sieves were used to prepare MMMs, with the aim to study the influence of the sole polymer phase on the gas transport properties [43,44]. Next, the molecular sieves were made fluorophilic as described in Section 2 above, and then were used for the preparation of membranes according to Scheme 1. In a typical procedure, the molecular sieves were homogeneously suspended in a polymeric solution by repeated cycles of mechanical stirring and ultrasound treatments (1). The resting suspension was poured into a vessel with a flat, levelled bottom (2), to allow for the evaporation of the solvent at room temperature (3). The residual solvent lingering in the peeled MMM was eliminated in a vacuum oven (4). The similar values in the density of the fillers (1.53–1.87 g·cm^−3^), of the solvents (about 1.7 g·cm^−3^) and of the polymers (1.7–2.0 g·cm^−3^) is probably the reason behind the absence of sedimentation problems during the slow evaporation step (3).

**Figure 4:**
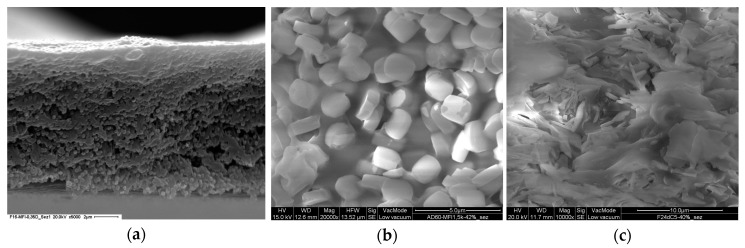
SEM cross-sections of PFP MMMs containing silicalite-1 crystals: (**a**) 30 wt% silicalite-1 (ca. 0.35 μm, AR 2) embedded in a Teflon AF1600 matrix; (**b**) 42 wt% silicalite-1 (1.5 μm, AR 3) embedded in a Hyflon AD60X matrix; (**c**) 40 wt% silicalite-1 (ca. 3 μm, AR 8) embedded in a Teflon AF2400 matrix. Reproduced from Reference [45] with permission. Copyright of John Wiley & Sons, Ltd.

Defect-free MMMs containing various amounts of silicalite-1 crystals of different size (from 0.08 to 1.5 μm) and aspect ratio (the ratio between the largest and the smallest dimension, AR in the following) were produced with Teflon AF1600, Teflon AF2400 and Hyflon AD60X, up to a volume fraction of 45% (Figure 4) [39,45].

**Figure 5:**
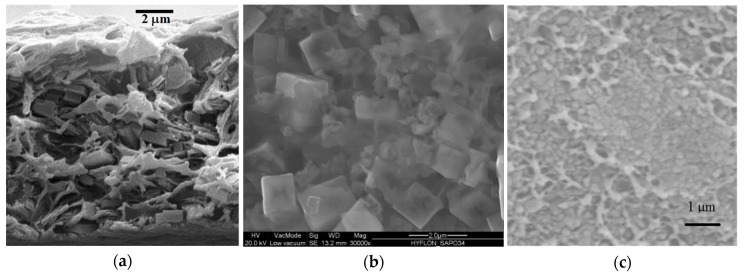
SEM cross-sections of Hyflon AD60X MMMs containing 35 v% SAPO-34 crystals of different size and aspect ratio: (**a**) 1.5 μm, AR 9; (**b**) 2 μm, AR 2; (**c**) 0.2 μm, AR 2. Reproduced from Reference [43] with permission. Copyright of the Korean Society of Industrial and Engineering Chemistry and Elsevier B.V.

**Figure 6:**
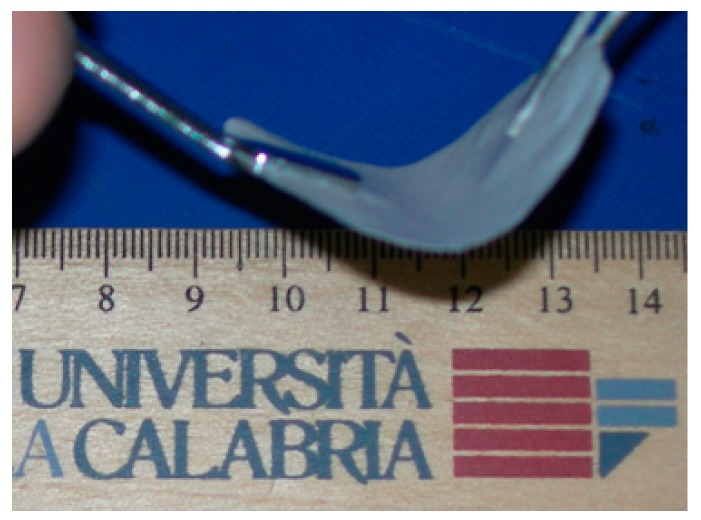
Bending of the Hyflon AD60X MMM containing 35 v% SAPO-34 crystals of 0.2 μm, AR 2. Reproduced from Reference [43] with permission. Copyright of the Korean Society of Industrial and Engineering Chemistry and Elsevier B.V.

Other MMMs were prepared by embedding 20–35% SAPO-34 crystals of different size (0.2–2 μm) and shape (AR 2–9), both permeable and impermeable, in Hyflon AD60X (Figure 5) [43]. Increasing fractions of molecular sieves made the membranes more rigid, but a good flexibility was still retained at 35 v% loading (Figure 6).

## 4. Transport Properties of Hydrophobic PFP/Molecular Sieve MMMs

### 4.1. PFP/Silicalite-1 MMMs

Silicalite-1 is a hydrophobic medium pore zeolite (5.4–5.6 Å) with channels delimited by 10-member rings of SiO_4_ units [46]. It is the siliceous form of ZSM-5 (structure topology MFI), with a three-dimensional pore network of straight and zigzag channels. It was chosen as the porous filler of PFPs because it can be easily prepared in a wide range of sizes and shapes, it is one of the most studied molecular sieves, with several gas sorption and mobility data available in the literature [47], and also because it only contains a modest amount of extra-framework cations and is hydrophobic [46]. Hydrophobicity represents an advantage for the modelling of gas transport because the large amount of water adsorbed by hydrophilic zeolites is an obstacle for the transport of gas, and in addition the uncertain amount of moisture, which rapidly adsorbs even during short expositions to the atmosphere, turns hydrophilic zeolite pore networks into ill-defined systems.

As a first important result, PFPs MMMs prepared with the fluorophilic silicalite-1 were all defect-free [39,45]: no gap was visible at the polymer-zeolite interface (Figure 4), and gas permeability data excluded the presence of defects. Transport features were dependent on the loading, and the size and the shape of the crystals.

**Figure 7:**
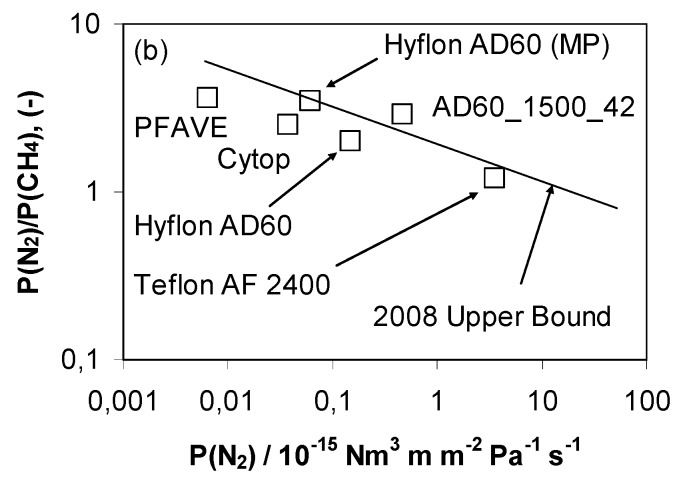
Selectivity vs. permeability plot for the N_2_/CH_4_ separation at 25 °C of perfluorinated polymers and a Hyflon AD60X MMM containing 42 w% (45 v%) Silicalite-1 (AD60_1500_42). Reproduced from Reference [45] with permission. Copyright of John Wiley & Sons, Ltd.

A Hyflon AD60X MMM containing 42 w% silicalite-1 of 1.5 μm size (Figure 4a,b) gave an interesting combination of permeability and N_2_/CH_4_ ideal selectivity, in excess of the 2008 upper bound [9] (Figure 7). The ideal CO_2_/CH_4_ selectivity was also improved in a similar way, getting closer to the 2008 upper bound for this gas pair [45]. These results were not expected, because the sizes of CO_2_, N_2_ and CH_4_ (Table 1) are all much smaller than the pore size of the MFI topology.

**Figure 8:**
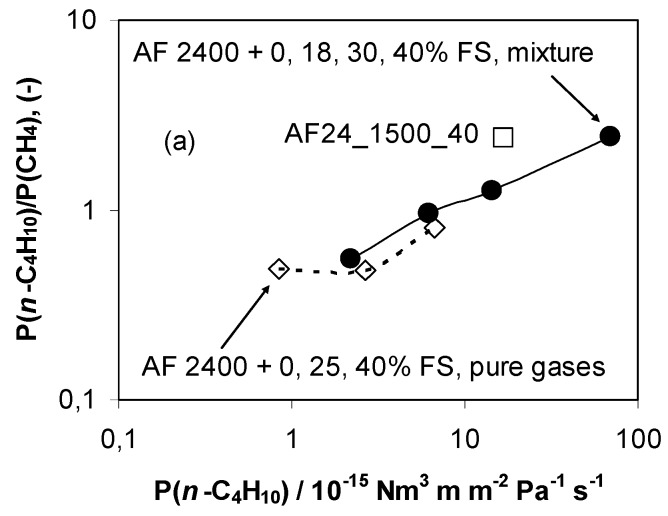
Selectivity vs. permeability plot of Teflon AF 2400 based hybrid membranes for the *n*-C_4_H_10_/CH_4_ pair at 25 °C: pure gas data of fumed silica filled AF2400 (◊: 0, 25 and 40%, Δp = 0.33–0.44 bar) from Reference [48]; pure gas data of membrane AF24_1500_40 (□, Δp = 1.00 bar); mixed gas data (2% n-butane feed, Δp = 11.2 bar) of fumed silica filled AF2400 (●: 0, 18, 30 and 40%), from Reference [48]; for all measurements the downstream pressure was atmospheric. Reproduced from Reference [45] with permission. Copyright of John Wiley & Sons, Ltd.

Teflon AF2400 MMMs were tested for the n-butane/CH_4_ separation [44]. Permeability and ideal separation factor of an MMM containing 40 w% silicalite-1 (1.5 μm size and AR 3, AF24_1500_40 in Figure 8) were higher than those of Teflon AF2400 hybrid membranes containing fumed silica [48]. The increase of polymer permeability upon the introduction of an impermeable filler (in that case fumed silica) was not expected. Positron annihilation (PALS) experiments demonstrated that the larger permeability of Teflon AF2400 hybrid membranes stems from free volume elements of larger size, probably caused by a looser packing of the polymer at the interface with silica [48]. Larger free-volume elements had been observed already for hybrid membranes containing fumed SiO_2_ of other glassy, high FFV polymers, such as poly(1-trimethylsilyl-1-propyne) (PTMSP) [49] and poly(4-methyl-2-pentyne) (PMP) [50], characterized by rigid backbones like Teflon AF2400. The decrease of the polymer density in Teflon AF1600 and Teflon AF2400 MMMs containing fumed silica was confirmed by detailed studies using the non-equilibrium lattice fluid (NELF) model, based on a non-equilibrium thermodynamics approach to glassy polymers [51,52]. The larger butane permeability measured when a porous filler is present with respect to the case in which fumed silica is incorporated indicated the active role of silicalite-1 in the transport of butane; also the larger C_4_H_10_/CH_4_ selectivity indicated the active participation of silicalite-1, in which the strongly adsorbed butane excludes methane [53].

When the AF24_1500_40 membrane was fed with equimolar butane-methane mixtures, a reversal of the selectivity from methane to butane on increasing the butane partial pressure (from 50 to 106 kPa at 25 °C) was observed. It is also interesting to note the advantages of a larger aspect ratio of the silicalite-1 crystals on the transport properties of the MMMs. The AF24_dC5_40 membrane, containing same loading (40 w%) of platy silicalite-1 crystals (3 μm, AR 8, Figure 4c), in all conditions afforded better butane/CH_4_ selectivity in the mixture experiments than the AF24_1500_40 membrane, with the same permeability [45].

Teflon AF1600 MMMs containing tiny silicalite-1 crystal (80 nm) were more permeable (He, H_2_, CO_2_, O_2_, N_2_ and CH_4_) than the polymer, and less selective. This behaviour might be the effect of a larger permeability of silicalite-1, of the increase of the FFV around the crystals, similarly to the Teflon AF2400/fumed silica MMMs, or else of the voids in the crystal aggregates contained in the MMMs.

Larger 350 nm silicalite-1 crystals, instead, distributed homogeneously in the Teflon AF1600 matrix (Figure 4a). A generalized decrease of gas permeability and a steady improvement of the sieving properties of the polymer were observed, as evidenced by the larger gas/CH_4_ ideal selectivity [45]. This effect was not expected, because silicalite-1 (pore size 5.4–5.6 Å) does not sieve small gas molecules (Table 1).

If the fillers are perfectly dispersed and do not interact with each other or with the matrix, the permeability of MMM is described by the Maxwell model [54] (Equation (5)):

(5) $$P^{MMM}=P^{M}\frac{P^{F}+2P^{M}-2\varphi \left(P^{M}-P^{F}\right)}{P^{F}+2P^{M}+\varphi \left(P^{M}-P^{F}\right)}$$

In Equation (5), *P^MMM^* is the gas permeability of the MMM, *P^F^* is the gas permeability of the dispersed phase (silicalite-1), *P^M^* is the gas permeability of the continuous phase (the polymer), and *φ* is the volume fraction of the dispersed phase. Since the experimental permeability of the polymer and of the MMMs were available, the gas permeability of silicalite-1 could be easily worked out. The different behaviour of the MMMs with silicalite-1 crystals of different size indicated that probably the filler does influence the physical state of the polymer. Therefore, it was decided to derive a theoretical permeability of the MMM from the gas permeability of filler and polymer, and to compare this value to the experimental results. The polymer permeability was readily measured, whereas the silicalite-1 permeability cannot be measured directly.

#### 4.1.1. Derivation of the Permeability of Silicalite-1

Care should be taken in the choice of the right gas permeability of a zeolitic molecular sieve from literature values. It has to be noted that in the literature a large scatter of the permeability values of different supported zeolitic membranes made of the same zeolite is found [47]. This happens because the gas permeability of zeolite membranes is affected by two major sources of error: (a) the presence of a variable amount of non-zeolitic pores, usually inter-crystalline space at the boundaries of the crystal domains [55], which act as non-selective defects increasing the membrane permeability, and (b) the presence of barriers to the transport of gas on the surface or in the interior of the crystals—crystal defects, interfaces in intergrowth crystals, presence of amorphous matter, moisture and/or coke [36,41,47,56,57,58,59,60,61,62,63,64,65,66,67,68,69,70,71]—which instead decrease gas permeability.

In practice, a more reliable method is to obtain the intrinsic gas permeability of a molecular sieve by combining solubility coefficients with the unrestricted intra-crystalline diffusion coefficients in Equation (2). Reproducible solubility of several gases in a variety of zeolites is available from experimental measurements; instead, the intra-crystalline gas diffusion coefficients are not, in general, available. The gas diffusion coefficients in molecular sieves, as obtained experimentally with different techniques, sometimes differ by three orders of magnitude or more. It is found that this scatter is the effect of surface and intra-crystalline barriers. The macroscopic techniques (e.g., sorption kinetics, chromatographic pulse methods) based on gas uptake usually measure a slower gas mobility than microscopic methods (such as pulsed field gradient nuclear magnetic resonance (PFG-NMR) and neutron scattering techniques), which are able to probe unrestricted diffusion inside the crystals [47]. Unfortunately, PFG-NMR and neutron scattering can only be used with some gases, and they are not readily available for investigation. Molecular dynamics (MD) simulations [47,72,73,74,75,76] represent a convenient way to evaluate the mobility of penetrants inside micro- and meso-porous media (the Maxwell–Stefan diffusion coefficient), in equilibrium conditions (self-diffusion), in the presence of concentration gradients, and with other co-adsorbed species.

The diffusion coefficients of CO_2_ and CH_4_ in silicalite-1 obtained with a transient pulse-response technique [77] were combined with the experimental solubility of the same gases [78] in Equation (2) in order to obtain the permeability coefficients in the molecular sieve [45].

#### 4.1.2. Modelling of the Transport Properties of Teflon AF1600/MFI MMMs

**Figure 9:**
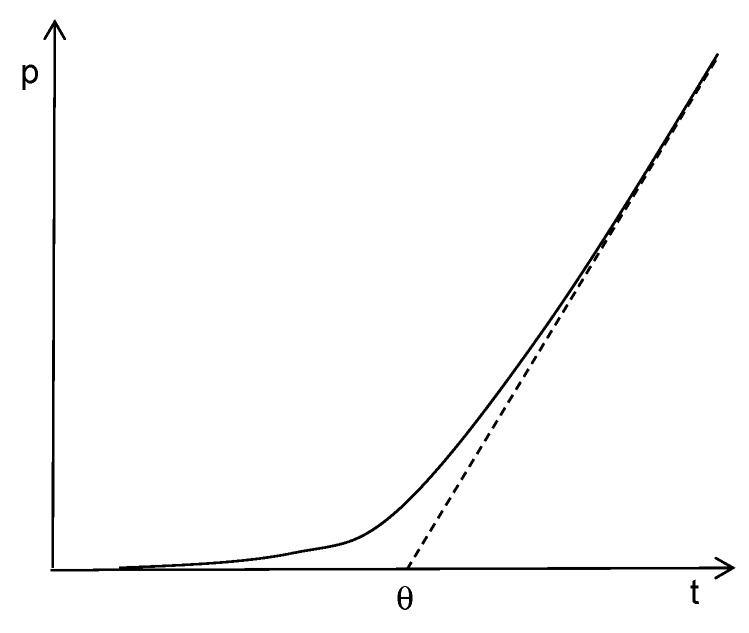
Increase of gas pressure (*p*) in the downstream receiving volume during a transient permeation experiment. *θ* is the so-called time-lag.

It turned out that silicalite-1 is definitely more permeable to CO_2_ and CH_4_ than Teflon AF 1600. As a consequence, the Maxwell Equation (5) predicted a larger permeability of the silicalite-1 containing MMMs with respect to the polymer. It was therefore surprising to discover that the experimental permeability of the MMM containing 30 w% of 350 nm silicalite crystals was smaller than the one of Teflon AF 1600 for both CO_2_ and CH_4_. The authors concluded that the silicalite permeability was strongly reduced by some barrier to the transport of gas at the surface or in the interior of the crystals [45]. In that work the average diffusion coefficients of gases through the Teflon AF 1600 MMMs was measured by means of the Daynes–Barrer time-lag method [79]. In this method the transient permeation behaviour before the attainment of the steady state is used to obtain average diffusion coefficients by means of Equation (6) below:

*D* = *l*^2^/6*θ* (6)

where *l* is the thickness of the membrane and *θ* (the time-lag) is the intercept of the pressure vs. time asymptotic line at long times with the time axis (Figure 9).

Silicalite-1 partially immobilizes the penetrant gases and due to its large adsorptive capacity, it acts like a sink for CO_2_ and CH_4_. Therefore, additional time is required to accumulate the excess penetrant before steady state can be reached [80,81,82,83,84]. This was probably the origin of the anomalous increase of the CO_2_ permeability observed after the apparent attainment of the steady state in a Teflon AF1600/silicalite-1 MMM [39]. The simplified treatment of the transient permeation experiments via Equation (6) underestimated the real, steady-state diffusion. Nevertheless, the time-lag diffusion coefficients of the two gases worked out with Equation (6) were higher in the MMM than through the Teflon AF 1600 polymer. Combined with the reduced permeability of the MMM, this circumstance indicated that the polymer phase in the MMM offered faster diffusion paths to gases, i.e., the polymer in the defect-free MMM was endowed with a larger free volume.

These earlier studies on PFP MMMs demonstrated that porous ceramic fillers, after the grafting of the perfluorinated tails, can be homogeneously dispersed in perfluorinated polymers. The presence of the filler disrupts the effective molecular packing of the polymer chains in the bulk, creating an interface with larger FFV. The increase of the ideal gas selectivity proved that such an interface is characterized by a good adhesion between the two phases. The macroscopic modelling of gas transport demonstrated also the presence of barriers for the transport of mass at the silicalite-1 crystals.

### 4.2. Hyflon AD60X/SAPO-34 MMMs

SAPO-34 is a family of silicon aluminium phosphate molecular sieves with the same pore topology of chabazite (CHA), a natural zeolite. In SAPO-34, large cavities are connected by narrow ports (3.8 Å) delimited by eight member rings of TO_4_ units [85]. Due to its small pore size, comparable to the diameter of methane (Table 1), SAPO-34 is very selective for the CO_2_/CH_4_ separation [86,87,88], and also displays interesting N_2_/CH_4_ selectivity [89].

On the basis of the experience with the PFP/silicalite-1 hybrids, new experiments were planned to make clear the importance for MMMs of interfacial polymer free volume and restricted diffusion in molecular sieves. Several Hyflon AD60X MMMs were prepared with different loadings (20–35 v%) of both permeable and impermeable SAPO-34 of different size and aspect ratios, with the aim to study in detail the effect of these variables on the final transport properties, and to isolate the behaviour of the polymer in the MMMs from the properties of the filler.

**Figure 10:**
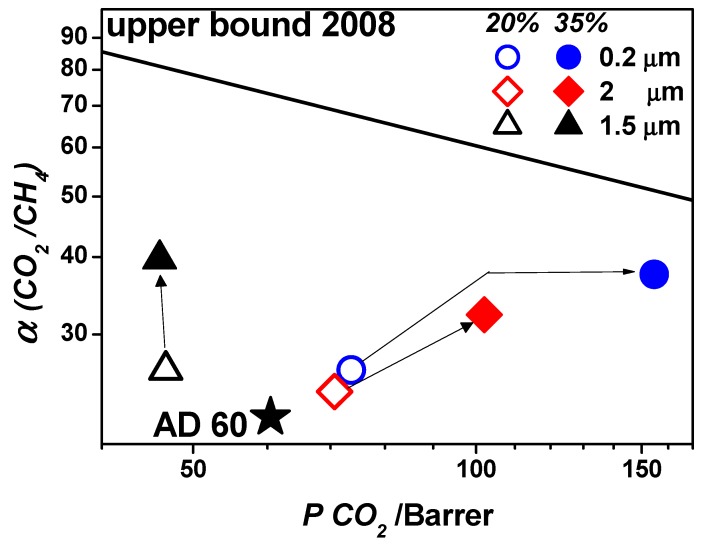
CO_2_/CH_4_ Robeson plot of Hyflon AD60X (AD60, black star) and of its MMMs containing calcined and permeable SAPO-34: tiles (1.5 μm, AR = 9, triangles), large (2 μm, AR = 2, diamonds), small (0.2 μm, AR = 2, circles); empty symbols 20 v% loading, full symbols 35 v% loading. 1 Barrer is equivalent to 10^−10^ cm^3^ (STP) cm cmHg^−1^·s^−1^·cm^−2^. Reproduced from Reference [43] with permission. Copyright of the Korean Society of Industrial and Engineering Chemistry and Elsevier B.V.

The MMMs prepared with the fluorophilic SAPO-34 were all defect-free [43]. No gap was visible at the polymer-zeolite interface (Figure 5), and gas permeability data (He, H_2_, N_2_, CH_4_, CO_2_) excluded the presence of defects. Transport features were dependent on the loading, the size and the shape of the crystals, as seen in the diagram of the Robeson’s plot for the CO_2_/CH_4_ gas pair (Figure 10).

In all cases the presence of SAPO-34 enhanced the selectivity of Hyflon AD60X for the CO_2_/CH_4_ separation. Thanks to the combination of the improved selectivity and of the plasticization resistance of the polymer, the MMMs may represent a viable option for the purification of natural gas containing large amounts of CO_2_. SAPO-34 afforded large selectivity gains with respect to the polymer, especially with 35% flat crystals of the largest aspect ratio (+81%). Inspection of Figure 5a revealed that the flat SAPO-34 crystals in this membrane were preferentially oriented with their largest dimensions about parallel to the membrane faces. The largest selectivity was accompanied by a decrease of permeability with respect to Hyflon AD 60X. Low aspect ratio SAPO-34 crystals at the same loading, resulted instead in an increase of both permeability and selectivity, with better results for the small (200 nm) SAPO-34. Since the Maxwell model—Equation (5)—predicts the same behaviour for the permeability of MMMs with the same loading of filler, irrespective of size and shape [54], it was evident that non-ideal effects were at work in Hyflon AD60X/SAPO-34 MMMs.

#### 4.2.1. Derivation of the Gas Permeability of SAPO-34

As in the case of the PFP membranes containing silicalite-1, the approach for the understanding of the underlying physical phenomena was the derivation of the unrestricted, intra-crystalline gas permeability of SAPO-34 (bulk values in the following), and its use in the macroscopic modelling of the transport phenomena in the MMMs [43].

The bulk permeability of He, H_2_, N_2_, CH_4_ and CO_2_ in SAPO-34 was assumed to be equal to the one of an all-silica zeolite with the same CHA topology. Bulk permeability was calculated as the product of solubility and diffusion (Equation (2)) [43] on the basis of in silico simulation results [90] of the pressure-dependent gas loading, and of the loading-dependent gas diffusivity in a SiO_2_ molecular-sieve with the CHA structure, in the fugacity interval 100–600 kPa.

#### 4.2.2. Modelling of the Transport Properties of Hyflon AD60X/SAPO-34 MMMs

The macroscopic model calculation of the gas transport in the MMMs varied according to the aspect ratio of the SAPO-34 crystals [43]. The resistance model of Cussler [91], derived for regular arrays of parallel ribbons (flakes) (Equation (7)), in which *AR* is the aspect ratio of the filler,

(7) $$P^{MMM}=\frac{P^{M}}{1-\varphi +\left\{1/\left[\left(1/\varphi \right)\left(P^{F}/P^{M}\right)+4\left(1-\varphi \right)/\left(AR^{2}\varphi^{2}\right)\right]\right\}}$$

approximates more closely the morphology of the MMMs containing oriented crystals of larger aspect ratio and is currently used to fit the experimental data in such cases [92,93].

The effective medium theory approach of Maxwell [54] was used for the MMMs containing crystals of smaller aspect ratio. Both the Cussler and the Maxwell models ignore interfacial effects, since they are based exclusively on the gas permeability of the unperturbed polymer, of the molecular sieve, on the aspect ratio of the filler (Cussler model only), and on their volume fractions.

**Figure 11:**
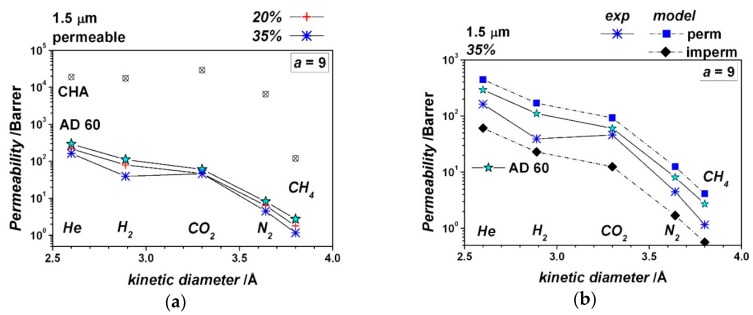
Experimental (continuous lines) and calculated (dash-dotted lines) gas permeability of Hyflon AD60X (AD60, stars) MMMs: 0%, 20% and 35% *v/v* of SAPO-34 tiles (1.5 μm, aspect ratio AR = 9). (**a**) MMMs and Hyflon AD60X experimental results; SAPO-34 (CHA, cross-hatched circles) permeability calculated after MD simulations [90] for the siliceous CHA structure. (**b**) Comparison of experimental (permeable tiles) and analytical results (Cussler model, both permeable and impermeable tiles), 35 v% loading. 1 Barrer is equivalent to 10^−10^ cm^3^ (STP) cm cmHg^−1^·s^−1^·cm^−2^. Reproduced from Reference [43] with permission. Copyright of the Korean Society of Industrial and Engineering Chemistry and Elsevier B.V.

The gas permeability of the MMMs containing flat crystals demonstrated the presence of barriers to the transport of mass at SAPO-34 [43]. The gas permeability of SAPO-34 is compared in Figure 11a with the experimental values of Hyflon AD60X (AD60) and of the MMMs with permeable SAPO-34 crystals of AR 9 (tiles). The unrestricted intra-crystalline permeability of the SAPO-34 crystals was much larger than that of the polymer, but the permeability of the MMMs was lower, with larger volume fractions (0.35 vs. 0.20) reducing the permeability even further. The experimental permeability did not obey the model prediction (Figure 11b), but it was larger than the prediction for an impermeable filler, indicating that SAPO-34 is permeable, and that its permeability is restricted. The presence of barriers for the transport of mass at zeolitic molecular sieves is the norm [47], although this is not acknowledged in most of the literature of MMMs. Diffusion experiments with infrared micro-imaging demonstrated the existence of surface barriers on SAPO-34 crystals, as well as the variability of surface barriers at different crystals from the same batch [64].

**Figure 12:**
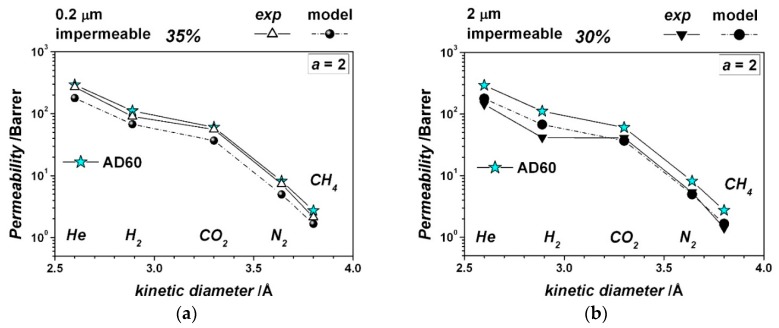
Experimental (continuous lines) and calculated (Maxwell model, dash-dotted lines) gas permeability of Hyflon AD60X (AD60, stars) MMMs with impermeable SAPO-34, aspect ratio AR = 2: (**a**) MMM containing small SAPO-34 (0.2 μm), 35% *v/v*; (**b**) MMM containing large SAPO-34 (2 μm), 30% *v/v*. 1 Barrer is equivalent to 10^−10^ cm^3^ (STP) cm cmHg^−1^·s^−1^·cm^−2^. Reproduced from Reference [43] with permission. Copyright of the Korean Society of Industrial and Engineering Chemistry and Elsevier B.V.

When SAPO-34 is impermeable, gas only permeates through the polymer phase, therefore the behaviour of MMMs prepared with same volume fractions of impermeable SAPO-34 gave valuable information on the state of Hyflon AD60X only in the MMMs [43]. Gas permeability for MMMs containing small (0.2 μm) impermeable SAPO-34 of aspect ratio 2 (Figure 12a) was larger than predicted by the Maxwell model, demonstrating the enlargement of the polymer free-volume at the interface with the filler. Small (0.2 μm) and large (2 μm) SAPO-34 of same aspect ratio were characterized by outer surface area of 39 and 5.3 m^2^·cm^−3^, respectively, therefore the volume of interfacial polymer in the MMMs containing small and large fillers, being roughly proportional to the outer surface area, was several times larger for the former. This circumstance was used to explain why the gas permeability of MMMs containing large impermeable SAPO-34 (Figure 12b) was close to the values predicted by the Maxwell model [43].

**Figure 13:**
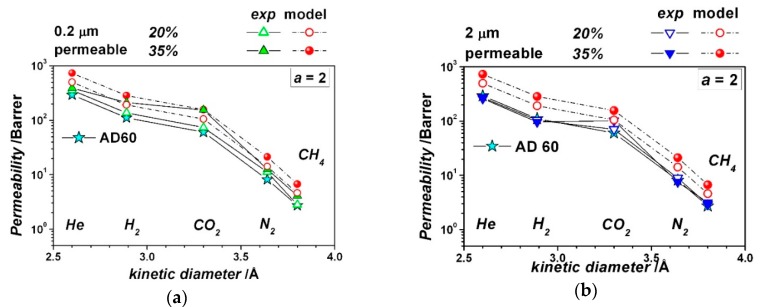
Experimental (continuous lines) and calculated (Maxwell model, dash-dotted lines) gas permeability of Hyflon AD60X (AD60, stars) MMMs with permeable SAPO-34, aspect ratio AR = 2: (**a**) MMMs containing small SAPO-34 (0.2 μm), 20 and 35% *v/v*; (**b**) MMMs containing large SAPO-34 (2 μm), 20 and 35% *v/v*. 1 Barrer is equivalent to 10^−10^ cm^3^ (STP) cm cmHg^−1^·s^−1^·cm^−2^. Reproduced from Reference [43] with permission. Copyright of the Korean Society of Industrial and Engineering Chemistry and Elsevier B.V.

Two distinct non-ideal effects, namely the increase of polymeric FFV at the interface with the filler and the barriers at SAPO-34, combined in the Hyflon AD60X/SAPO-34 MMMs [43]. The experimental gas permeability of MMMs containing small (0.2 μm) permeable SAPO-34 of aspect ratio 2 (Figure 13a) was comparable to the prediction of the Maxwell model, because the larger interfacial polymer free-volume compensated the effect of the barriers at SAPO-34. Instead, the experimental gas permeability of MMMs containing large impermeable SAPO-34 (Figure 13b) was lower than the values predicted by the Maxwell model, because in this case the quantity of the interfacial polymer of higher free volume was minimal [43].

**Figure 14:**
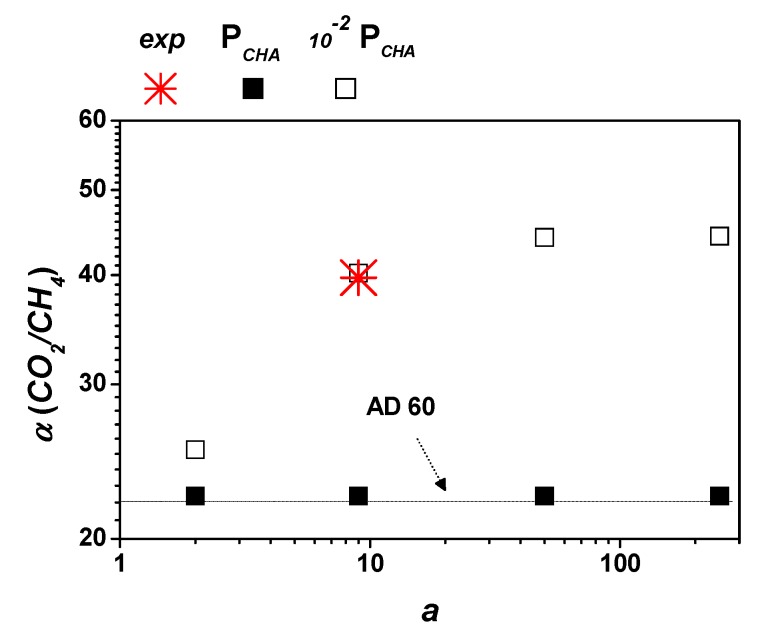
Experimental (asterisk) and calculated (squares, Cussler model) CO_2_/CH_4_ ideal selectivity of Hyflon AD60X MMMs containing 35% *v/v* of SAPO-34 of different aspect ratios (*a* on the abscissa). The constant selectivity of the polymer [43] is reported as a full line for comparison; full squares refer to the ideal selectivity of MMMs calculated after MD simulations for the siliceous CHA structure [90]; open squares refer to the ideal selectivity of MMMs calculated after MD simulations for the siliceous CHA structure [90], when the derived, unrestricted gas permeabilities used for the model calculation are divided by 100. Reproduced from Reference [43] with permission. Copyright of the Korean Society of the Industrial and Engineering Chemistry and Elsevier B.V.

The Maxwell (Equation (5)) and the Cussler (Equation (7)) models were used to calculate the expected ideal CO_2_/CH_4_ selectivity for Hyflon AD60X MMMs containing 35% *v/v* of SAPO-34 with different aspect ratios [43]. If mass transport in SAPO-34 were not restricted by the presence of barriers (filled squares in Figure 14), then the gain in selectivity with respect to the polymer would be irrelevant. If instead the restricted diffusion of mass reduced the effective average permeability of SAPO-34 by two orders of magnitude (empty squares in Figure 14), then the selectivity raised asymptotically to about 45 for AR ~50. This finding demonstrated that very selective fillers do not improve the MMM selectivity if they are too permeable compared to Hyflon AD60X, as predicted in the literature [92,93]. Another interesting information was that most of the selectivity gain could be obtained already with aspect ratio 9 (Figure 14).

**Figure 15:**
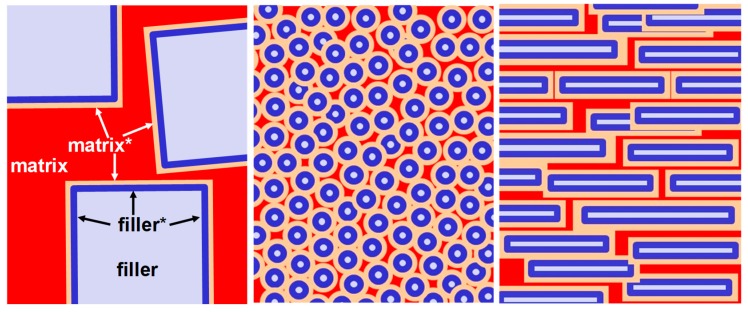
Schematic representation of three Hyflon AD60X MMMs containing SAPO-34 crystals of different size and shape (large, small, and tiles in the text) in the four resistance models (see text). “filler*” (dark-blue) and “matrix*” (light-orange) denote, in all of the three illustrations, the modified diffusion layers of SAPO-34 and Hyflon AD60X, respectively.

The modelling of gas transport in Hyflon AD60X/SAPO-34 MMMs with the effective medium approach (Cussler and Maxwell models) made it clear that, besides the unperturbed (bulk) polymer and filler, a region of interfacial polymer and some barrier at SAPO-34 must be considered. Starting from this evidence, Di Maio et al. [44] introduced a new four phase approach of MMMs which explicitly takes into account, besides the matrix and filler bulk phases, the interfacial polymer phase (“matrix*” in Figure 15) and the barriers at SAPO-34. For the sake of simplicity, the latter were concentrated into a skin of reduced permeability encapsulating the core with unrestricted mobility (“filler*” and “filler”, respectively, in Figure 15).

The new four-phase approach was implemented for He, H_2_, N_2_ and CO_2_ permeation in Hyflon AD60X/SAPO-34 MMMs in two ways: by means of the effective medium analytical formula (4M Maxwell model), and through the finite element solution (FEM) of the transport differential equations [44].

**Figure 16:**
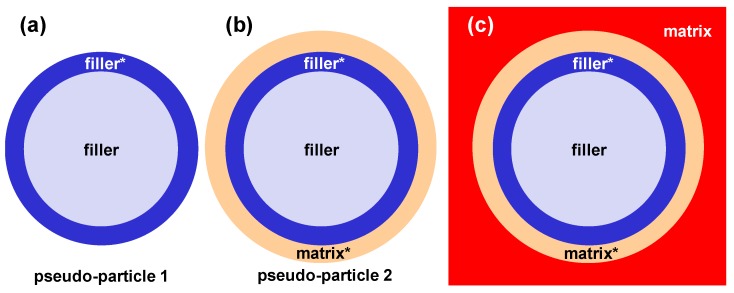
The three frames of the 4M Maxwell model procedure: (**a**) pseudo-particle 1 composed of bulk filler and modified filler; (**b**) pseudo-particle 2, composed of pseudo-particle 1 and modified matrix; (**c**) the whole mixed-matrix membrane, composed of pseudo-particle 2 and bulk matrix.

The 4M Maxwell model was inspired by a previous three-phase Maxwell model introduced by Koros and co-workers [94,95], and the procedure for the workout of the MMM permeability consisted in three steps. First the SAPO-34 crystals were treated as pseudo-particles made of a very permeable core with a poorly permeable skin (“filler” and “filler*”, pseudo-particle 1 in Figure 16a). The permeability of pseudo-particle 1 was calculated by means of the Maxwell Equation (5). Second, a new pseudo-particle made of the whole filler (pseudo-particle 1) surrounded by the modified polymer (pseudo-particle 1 plus “matrix*”, pseudo-particle 2 in Figure 16b) was considered, and its permeability was calculated by using again the Maxwell Equation (5). Finally, the MMM permeability was calculated by combining the permeability of the pseudo-particle 2 with the permeability of the bulk polymer (pseudo-particle 2 plus “matrix”, Figure 16c). The unknowns regarding the polymer (i.e., the permeability and the thickness of the interfacial polymer, “matrix*” in Figure 16) were removed in part by applying the Maxwell Equation (5) to the experimental permeability data of MMMs containing same volume fractions of identical, but impermeable, molecular sieve particles [95]. The unrestricted permeability of SAPO-34—i.e., the permeability of the SAPO-34 core, “filler” in Figure 16—was calculated as described in Section 4.2.1. More details on the formulations of the relevant analytical equations can be found in the Supplementary Materials of Reference [44]. It has to be noted that the permeability and the thickness of the interfacial polymer surrounding the particles of filler (“matrix*”) are not independent of each other, as well as the thickness and the permeability of the barrier skin at SAPO-34 (“filler*”). From the mathematical point of view, in both cases a larger difference in the permeability from the value of the bulk corresponds to a thinner layer, and vice versa. A thickness of 20 nm for the modified polymer layer (“matrix*” in Figure 15) was adopted in order to avoid the overlapping of vicinal layers in the FEM simulation (vide infra), because this length was just less than half of the average distance among the smallest (200 nm) crystals at maximum (35%) volumetric loading. A 20-nm thickness was also chosen for the barrier layer at SAPO-34 (“filler*” in Figure 15). A recent theoretical work on a comparable zeolite—SAS topology, made of large cavities connected by 8-member rings of TO_4_ units—found a thickness of surface barriers of the same order of magnitude, i.e., 3, 7 and 14 nm for H_2_, CO_2_ and CH_4_, respectively [96]. For simplicity, gas independent filler* thickness was considered in the 4M model calculation.

**Figure 17:**
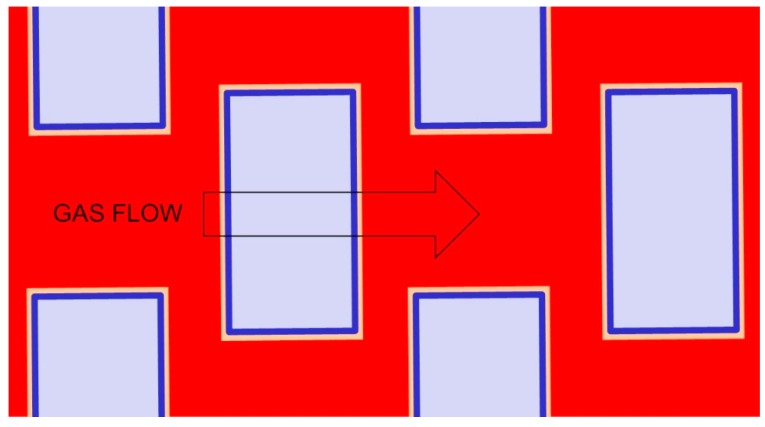
Bi-dimensional scheme of the body-centred cubic arrangement of oriented SAPO-34 particles built by the application software for the representation of the four-resistance model.

Numerical simulation of the gas permeation through composite membranes was carried out by solving the finite-element approximation of the partial differential equations governing the relevant solution/diffusion processes [44], using the commercial software COMSOL Multiphysics [97]. In order to simplify the computational model, a body-centred cubic arrangement of oriented filler particles was built to generate symmetrical properties of the system (Figure 17).

Pure gas diffused continuously through the distinct matrix and filler domains. Diffusion was expressed in terms of *C_i_,* the concentration of species *i*. The following equations:

(8) $$D_{ij}\left(\frac{\partial^{2}C_{i}}{\partial x^{2}}+\frac{\partial^{2}C_{i}}{\partial y^{2}}+\frac{\partial^{2}C_{i}}{\partial z^{2}}\right)=0$$

in which the diffusivity coefficient *D_ij_* of species *i* in material *j* is assumed uniform and isotropic, were solved. A concentration jump at the matrix/filler interface formed due to the different gas solubility. At the interface boundaries both uniform mass flux and the following equilibrium condition were applied:

(9) $$\frac{C_{i}{}^{I}}{S_{i}{}^{I}}=\frac{C_{i}{}^{II}}{S_{i}{}^{II}}$$

where *S_i_* is the solubility of species *i* and the superscripts denote the two solid phases, *I* and *II*, in contact.

**Figure 18:**
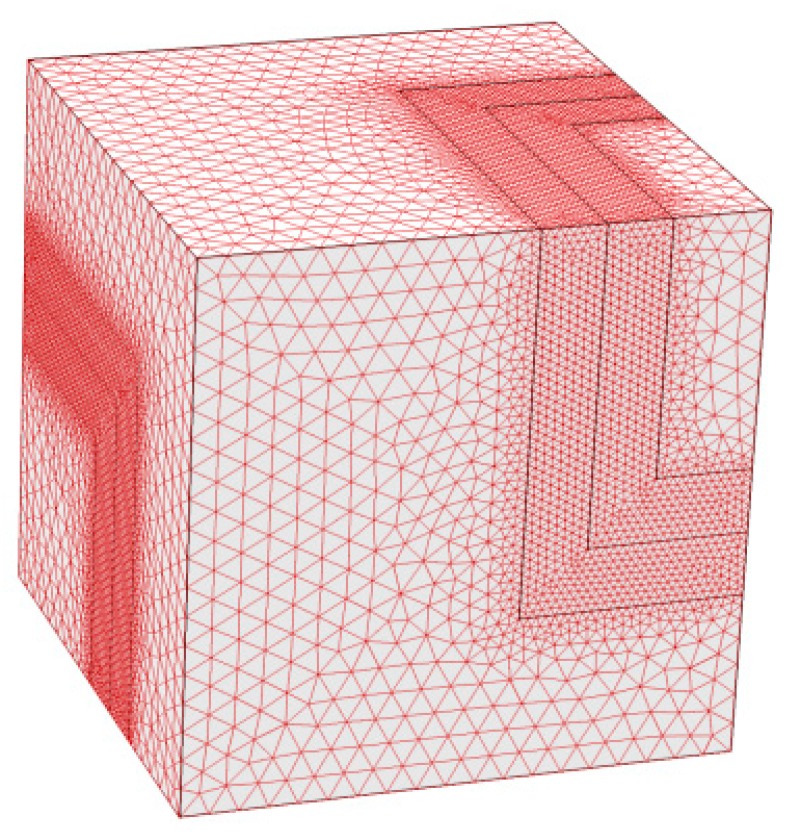
Geometry and discretization grid of the computational cell for a system containing 0.2 μm SAPO-34, AR = 2, volume fraction 0.20. Inlet is from the left front face and outlet from the right rear face. On the other four faces symmetry conditions apply. Reproduced from Reference [44] with permission. Copyright of Elsevier B.V.

Computational grids (Figure 18) were built by using the application software.

The inputs of the FEM simulations were: (i) SAPO-34 bulk diffusivity and solubility data from simulations (see Section 4.2.1.); (ii) the experimental polymer permeability; (iii) the polymer gas diffusivity from the literature [98]; (iv) the permeability of the ten different MMMs containing permeable and impermeable molecular sieves. By imposing 20 nm as the thickness of each modified phases (“matrix*” and “filler*” in Figure 15), the permeability ratios between the pristine and the modified phases were optimized for each gas by a least square iterative minimization of the discrepancies between calculated and experimental permeability. The properties of the modified polymer layer (“matrix*”) were obtained in a first optimization step using the data subset including only MMMs incorporating impermeable fillers. Then the results of the former step were used to focus on the properties of the modified SAPO-34 layer (“filler*”). Additional details on the numerical procedure can be found in Reference [44]. The FEM simulations indicated that the permeability in the high FFV polymeric interface (“matrix*”) increased of 2.6 ± 0.3 times, whereas instead the permeability of the barrier skin of SAPO-34 (“filler*”) decreased by a factor ranging from 3.5 × 10^−5^ to 1.1 × 10^−4^ (Maxwell 4M), or from 3.8 × 10^−5^ to 4.3 × 10^−4^ (FEM) [44].

**Figure 19:**
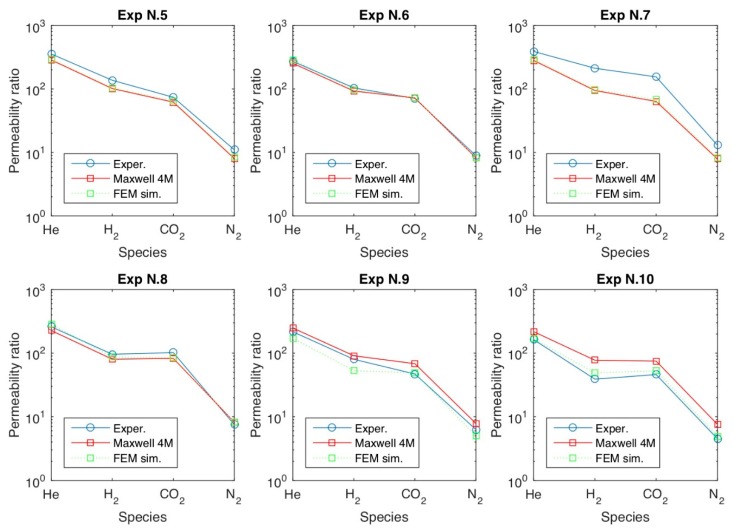
Comparison between the experimental permeability of Hyflon AD60X/SAPO-34 MMMs and their predictions based on the 4-phase approach according to the Maxwell model and the FEM simulations for permeable particles, cases No. 5–10 in Table 2. Reproduced from Reference [44] with permission. Copyright of Elsevier B.V.

By the above assumptions, the results of both FEM simulations and macroscopic 4M Maxwell model compared well with the experimental data (Table 2 and Figure 19).

Not surprisingly, the best fit was found with the MMMs containing the large fillers (2 μm, AR 2, exp. 6, 8) in which the relative importance of the interfacial effects was minimal (Table 2 and Figure 19). In the case of small SAPO-34, both methods underestimated of the same quantities the permeability of the MMMs (Figure 19, 0.2 μm, AR 2, exp. 5,7), probably due to the formation of percolation paths connecting the modified polymer phase (“matrix*” in Figure 15) in real membranes. The FEM simulation was able to reproduce in a better way the data of the MMMs containing oriented flat crystals (Figure 19, tiles, 1.5 μm, AR 9, exp. 9, 10), because the large aspect ratio and the orientation of the filler were implicit in the model. Instead, the macroscopic 4M Maxwell model, based on spherical particles, showed a worse fit, overestimating the real permeability. Notice that the predictions of the 4M Maxwell model are satisfactory even at relatively high-volume fractions (35%) of the filler.

The overall agreement of the experiments with both the macroscopic 4M Maxwell modelling and the FEM simulations is excellent if we consider the simplifying assumptions adopted, and namely: (i) the constant, average values of solubility and diffusion coefficients for polymer and filler bulk; (ii) the uniform constant features of the surface barrier for all of the three different samples of SAPO-34.

**Figure 20:**
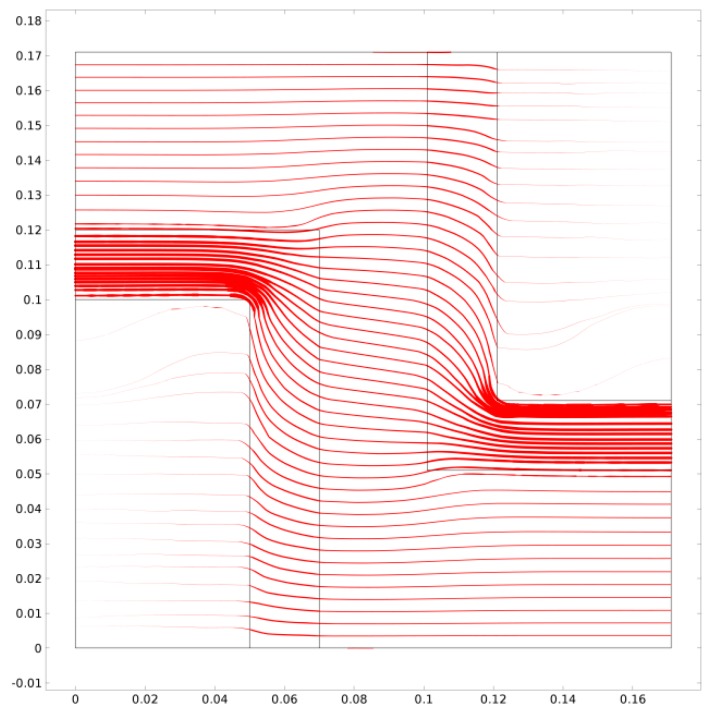
Experiment No. 5 (SAPO 0.2 μm size, volume fraction 20%, AR = 2, see also Table 2 and Figure 18): streamlines of the CO_2_ flow in a horizontal plane at half of the cell height. The streamlines are initiated at 50 points uniformly distributed on an imaginary vertical line located halfway along the transport direction and then reconstructed back and forth; their line thickness is proportional to the flow magnitude and axis units are in μm. Reproduced from Reference [44] with permission. Copyright of Elsevier B.V.

The major advantages of the FEM simulation consist in the quantification of the contributions of the different phases to the overall gas transport and in the possibility to visualize the concentration profiles in a unit cell [44] as well as the streamlines of the gas (Figure 20). The FEM simulation clearly indicated the lesser contribution of SAPO-34 to the overall transport in the MMMs containing small crystals of modest aspect ratio, whereas the predominant role of high free volume polymer led to high permeability. Instead SAPO-34 contribution to transport was maximal when oriented crystals of large aspect ratio were present, consistent with the lesser permeability and the larger selectivity of the MMMs.

### 4.3. Remarks and Perspectives on the Modelling of Transport in MMMs

The lesson learned from the study of the PFP/molecular sieve systems is that the macroscopic modelling of the transport properties of MMMs requires the understanding of the underlying physical phenomena. If we had only prepared MMMs with the high aspect ratio SAPO-34 (triangles in Figure 10) we might have argued about the prevalence either of a rigidified polymer layer around the filler, or of a reduced permeability region within the sieve surface [35]: the possibility of a high FFV polymer shell surrounding the filler would have not been considered.

The physical state of polymers at MMM interfaces should be investigated in depth to probe the effect of fillers on free volume, adsorption properties, swelling ability, ageing rate. Positron annihilation lifetime spectroscopy (PALS) [99], ^129^Xe NMR [17], and the NELF model [52] applied to MMMs containing impermeable fillers look like valuable tools for this purpose.

It has to be noted that the new four-phase approach is not restricted to the modelling of PFP MMMs, since it can be applied whenever two non-ideal phenomena are present, including those cases in which a rigidified polymer layer is observed. Besides the consistent interpretation of the physical phenomena, an adequate modelling of the transport properties of real systems allows predictive capabilities to guide the preparation of better MMMs.

Crystalline porous materials, in real life, contain defects and are far from ideal. The modelling of mass transport in MMMs needs theoretical and experimental studies of diffusion in the fillers, both ideal and defective. The nature and the effects of the barriers to mass transport need to be studied in depth. In MMMs containing extremely thin, exfoliated layers, either zeolitic molecular sieves, porous graphenes, MOFs or others, the transport properties of the bulk materials may be altered to a large extent, and interfacial effects become of primary importance.

## 5. Summary and Conclusions

Grafting perfluorinated tails on the outer surface of zeolitic molecular sieves improved the surface permeability and allowed the preparation of defect-free MMMs with hydrophobic and amorphous PFPs.

Silicalite-1/Hyflon AD60X MMMs displayed some improvement of the CO_2_/CH_4_ and N_2_/CH_4_ ideal selectivity with respect to the polymer. The improvements were not expected, because the pores of silicalite-1 are not able to discriminate the above gases on the basis of size. Methane is more permeable than *n*-butane in Teflon AF2400 membranes, but the presence of silicalite-1 in Teflon AF2400 MMMs was able to revert the selectivity in favour of *n*-butane, probably because of the combined effects of a larger interfacial FFV and of the butane-selective molecular sieve. Other things being equal, large aspect ratio silicalite-1 increased the *n*-butane/CH_4_ selectivity even further.

The gas transport properties of Teflon AF1600 MMMs containing silicalite-1 crystals were strongly dependent on the size of the filler. Small crystals added permeability (He, H_2_, CO_2_, O_2_, N_2_ and CH_4_) to the polymer with less selectivity, whereas larger crystals induced a reduction of permeability and an increase of the gas/CH_4_ selectivity. In order to clarify this puzzling situation, a change of paradigm was introduced: instead of calculating the permeability of the filler from the performance of the MMMs through the Maxwell model (Equation (5)), the permeability of the filler was obtained from the gas solubility and diffusion, using this value to predict the performance of an ideal MMM through Equation (5). The comparison of the predicted vs. the experimental permeability (CO_2_ and CH_4_) and the evaluation of the experimental diffusion coefficients (time lag method) indicated the presence of two different non-ideal effects in the Teflon AF1600/silicalite-1 MMMs: a polymer allowing larger gas mobility, i.e., a larger polymer FFV, and a much less permeable filler, i.e., the presence of barriers to the transport of mass at silicalite-1.

A new set of Hyflon AD60X MMMs were prepared with three different SAPO-34 crystals, of different size and aspect ratio, both permeable and impermeable. All of the MMMs where characterized by improvements in the ideal CO_2_/CH_4_ selectivity, but the permeability was strongly influenced by the size and the shape of SAPO-34. The large aspect ratio crystals preferentially oriented parallel to the plane of the membrane and induced a reduction of the polymer permeability. The filler with modest aspect ratio, instead, determined an increase of the permeability, with the smaller SAPO-34 crystals resulting in the larger gain. The analysis of the transport properties of the MMMs subset containing impermeable SAPO-34 clarified that Hyflon FFV had increased. As in the case of silicalite-1, the permeability of SAPO-34 was obtained from independent solubility and diffusivity values and used to predict the performance of the MMMs through Equation (5). The predicted MMMs permeability was higher than the experimental results, evidencing the presence of barriers to the transport of mass at SAPO-34. On the basis of the experimental evidences, the transport properties of the MMMs were modelled through a new four phase approach, which explicitly considered the pristine two phases, i.e., the unperturbed, bulk polymer and SAPO-34, and two interfacial phases, namely the perturbed, high FFV polymer, and a superficial skin of the crystals, concentrating all of the additional resistance to the transport of mass (the barrier). The four-phase macroscopic modelling, implemented both via an extension of the Maxwell analytical method (4M Maxwell model) and by means of a FEM numerical simulation, nicely reproduced the experimental results. The new four-phase approach lends itself to interpret the transport data of those MMMs evidencing two simultaneous non-ideal behaviours.

The above pioneering works clearly show: the feasibility of PFP/molecular sieve MMMs and their interesting properties for gas separation of technological relevance; the complexity and the competitive character of the processes at work in MMMs; the adequacy of four-phase models for the description of permeation in MMMs.

## Figures and Tables

**Table 1:** Kinetic diameter (d_k_) [25] and critical temperature (T_c_) [26] of six gases.

Gas	He	H_2_	CO_2_	O_2_	N_2_	CH_4_
d_k_/Å	2.6	2.89	3.3	3.46	3.64	3.80
T_c_/K	5.2	33.18	304.20	154.58	126.19	190.6

**Table 2:** Features of the Hyflon AD60X MMMs containing permeable SAPO-34. Modified from Reference [44] with permission. Copyright of Elsevier B.V.

Experiment No.	5	6	7	8	9	10
Crystal longest size, μm	0.2	2	0.2	2	1.5	1.5
Particle Aspect Ratio, -	2	2	2	2	9	9
Particle volume fraction, -	0.20	0.20	0.35	0.35	0.20	0.35

## List of Symbols and Acronyms

4M	four-phase Maxwell model
*α*	permeability selectivity, -
*α_S_*	solubility selectivity, -
*α_D_*	diffusion selectivity, -
*φ*	volume fraction, -
*θ*	time-lag, s
*AR*	aspect ratio, -
*Barrer*	permeability unit, 10^−10^ cm^3^ (STP) cm cmHg^−1^·s^−1^·cm^−2^
*BET*	Brunauer, Emmett and Teller method for the determination of the surface area
*C*	concentration, mol m^−3^ or cm^3^(STP) cm^−3^
*CHA*	framework type code of zeolite chabasite
*Cytop* ^®^	poly(perfluoro-4-vinyloxy-1-butene)
*D*	diffusivity, m^2^/s
*d_k_*	kinetic diameter, Å
*FEM*	finite element numerical modelling
*FFV*	fractional free volume, -
*Hyflon^®^ AD*	co-polymers of tetrafluoroethylene and perfluoro-4-methoxy-1,3-dioxole
*J*	flux, mol/(m^2^·s) or cm^3^(STP)/(cm^3^·s)
*l*	membrane thickness, μm
*MD*	molecular dynamics
*MFI*	framework type code of zeolite ZSM-5
*MMM*	mixed matrix membrane
*MOF*	metal organic framework
*Nafion^®^*	copolymers of tetrafluoroethylene and sulfonic acid terminated perfluorovinyl ethers
*NELF*	non-equilibrium lattice fluid
*NMR*	nuclear magnetic resonance
*p*	pressure, Pa
*P*	permeability, Barrer or Nm^3^·m·m^−2^·Pa^−1^·s^−1^
*P^F^*	permeability of the dispersed phase, Barrer or Nm^3^·m·m^−2^·Pa^−1^·s^−1^
*P^M^*	permeability of the continuous phase, Barrer or Nm^3^·m·m^−2^·Pa^−1^·s^−1^
*P^MMM^*	permeability of the MMM, Barrer or Nm^3^·m·m^−2^·Pa^−1^·s^−1^
*PALS*	positron annihilation lifetime spectroscopy
*PFG-NMR*	pulsed field gradient NMR
*S*	solubility, mol·m^−3^·Pa^−1^ or cm^3^(STP)·cm^−3^·Pa^−1^
*T_c_*	critical temperature, K
*T_g_*	glass transition temperature, K
*PFP*	perfluorinated polymer
*PIM*	polymer of intrinsic microporosity
*SAPO-34*	silicon aluminium phosphate of chasbasite (CHA) structure topology.
*SEM*	scanning electron microscopy
*Silicalite-1*	pure SiO_2_ zeolite of ZSM-5 (MFI) structure topology
*Teflon^®^*	polytetrafluoroethylene
*Teflon^®^ AF*	co-polymer of tetrafluoroethylene and perfluoro-2,2-dimethyl-1,3-dioxole
*STP*	standard temperature and pressure
